# Time Integrated Flux Analysis: Exploiting the Concentration Measurements Directly for Cost-Effective Metabolic Network Flux Analysis

**DOI:** 10.3390/microorganisms7120620

**Published:** 2019-11-27

**Authors:** Rui M. C. Portela, Anne Richelle, Patrick Dumas, Moritz von Stosch

**Affiliations:** 1 Process Systems Biology and Engineering Center of Excellence, Technical Research and Development, GSK Biologicals, 1330 Rixensart, Belgium; ruimiguel.x.correiaportela@gsk.com (R.M.C.P.); anne.x.richelle@gsk.com (A.R.); 2 Cell and Viral Drug Substance, Technical Research and Development, GSK Biologicals, 1330 Rixensart, Belgium; patrick.dumas@gsk.com

**Keywords:** Metabolic Flux Analysis, Flux Balance Analysis, Flux Variability Analysis, Metabolic modeling, Specific Flux Estimation, Specific Rate Estimation, Time Integrated Flux Analysis

## Abstract

Background: Flux analyses, such as Metabolic Flux Analysis (MFA), Flux Balance Analysis (FBA), Flux Variability Analysis (FVA) or similar methods, can provide insights into the cellular metabolism, especially in combination with experimental data. The most common integration of extracellular concentration data requires the estimation of the specific fluxes (/rates) from the measured concentrations. This is a time-consuming, mathematically ill-conditioned inverse problem, raising high requirements for the quality and quantity of data. Method: In this contribution, a time integrated flux analysis approach is proposed which avoids the error-prone estimation of specific flux values. The approach is adopted for a Metabolic time integrated Flux Analysis and (sparse) time integrated Flux Balance/Variability Analysis. The proposed approach is applied to three case studies: (1) a simulated bioprocess case studying the impact of the number of samples (experimental points) and measurements’ noise on the performance; (2) a simulation case to understand the impact of network redundancies and reaction irreversibility; and (3) an experimental bioprocess case study, showing its relevance for practical applications. Results: It is observed that this method can successfully estimate the time integrated flux values, even with relatively low numbers of samples and significant noise levels. In addition, the method allows the integration of additional constraints (e.g., bounds on the estimated concentrations) and since it eliminates the need for estimating fluxes from measured concentrations, it significantly reduces the workload while providing about the same level of insight into the metabolism as classic flux analysis methods.

## 1. Introduction

The analysis of metabolic fluxes in quasi-steady state has helped to increase the understanding of cell metabolism [1,2,3,4]. Depending on the number of measured concentrations, the considered metabolic network as well as the objective of the analysis, different methods can be applied, the most widely adopted ones being the Metabolic Flux Analysis (MFA) [5,6], Flux Balance Analysis (FBA) [7,8] and Flux Variability Analysis (FVA) [9].

For the analysis of experimental data with these flux analysis methods, estimation of the fluxes that cross the cell boundary from the concentration measurements is required. This flux estimation can be accomplished in two different ways: differential or integral [10,11].

For the differential way, most commonly and in best practice, an arbitrary time dependent function is fitted to approximate the measured concentrations [12,13,14] and then, the derivative of the function with respect to time is computed. The computed derivative is then divided by the biomass concentration (either using the measured concentration or the function that approximated the biomass measurements). The computation of the specific rates in this way is known to be a mathematically ill-conditioned inverse problem, i.e., potential errors in the measurement of the concentrations are propagated and amplified. Only in case certain criteria for the number of measurements and the error of the measurements are met, are the errors in the estimated rates acceptable [12].

For the integral way, there exist, in principal, two possibilities to obtain values for the fluxes: (1) Kinetic rate functions could be defined for the specific fluxes that are comprised in the extracellular material balances (i.e., the mass balances that describe the evolution of the extracellular compounds). The parameters contained in these functions can then be fitted by numerically integrating the material balance and minimizing the residual between the predicted and measured concentration values. However, the formulation of the kinetic rate functions is not always straightforward. (2) The experiment can be divided into distinct phases, during which the changes in flux values are assumed to be negligible (e.g., during exponential growth) [15]. For each phase, the macroscopic material balance can be numerically or analytically integrated and the residuals between the measured and predicted concentrations can be minimized by adapting the flux values. However, the partition of the experiment into distinct phases is not always easy and the assumption that the flux values do not vary during the phase does not always hold.

In what follows, a new approach for flux analysis is proposed that adopts the time-integrated form of the macroscopic material balances (without any assumption on the flux values or the kinetic functions) to estimate the amount of material that is transformed by the intracellular pathways. Since the amount of material that is transformed is tantamount to the time integrated fluxes, the proposed approach is coined time integrated flux analysis. This approach follows the same idea as set forward in the parameter estimation approach described in Liu and Gunawan [16]. However, in the proposed approach, the amount of transformed material is estimated rather than model parameters. The approach is adopted for MFA, FBA and FVA as well as sparse FBA. The MFA adaptation is applied to two simulated examples assessing the methods capability with respect to different levels of errors in the concentration measurements and different numbers of samples taken during the experiment as well as the impact of network redundancy and reaction reversibility. The sparse FBA adaptation is applied to an experimental example to showcase the practical relevance of the approach.

## 2. Methods

The extracellular Equation (1) and intracellular Equation (2) material balances provide the backbone to the proposed method and read:

(1) $$\frac{dc_{ex}\cdot V}{dt}=q\cdot x\cdot V+u,$$

(2) $$\frac{dc}{dt}=S\cdot v+S_{q}\cdot q-\mu \cdot c,$$

where $c_{ex}$ is a vector of extracellular concentrations that also comprises the biomass concentration x, V is the volume of the broth, q is a vector of specific rates (also comprising the specific biomass growth rate μ), u is a vector of compound specific feeding rates (in case of fed-batch or continuous operation), c is a vector of intracellular concentrations, v is a vector of intracellular fluxes, and S and $S_{q}$ are the stoichiometric matrices, which are obtained from the metabolic network. In case of intracellular balance, common assumptions are those of quasi steady-state ($\frac{dc}{dt}=0$) and q and v being much greater than μ·c, whereby Equation (2) can be simplified to.

(3) $$0=S\cdot v+S_{q}\cdot q$$

The aim is to reformulate this equation such that the concentrations measurements can be used directly. Multiplication with x·V·dt and integration yields:

(4) $$0=S\cdot \int_{t_{0}}^{t}v\cdot x\cdot V\cdot dt\;+S_{q}\cdot \int_{t_{0}}^{t}q\cdot x\cdot V\cdot dt$$

where the stoichiometric matrices can be moved in front of the integral since the coefficients are time-invariant. The second integral term is identical to one of the terms in the integrated version of the extracellular material balance (Equation (1)):

(5) $$\int_{t_{i}}^{t_{j}}q\cdot x\cdot V\cdot dt=c_{ex}\left(t_{j}\right)\cdot V\left(t_{j}\right)-c_{ex}\left(t_{i}\right)\cdot V\left(t_{i}\right)-\int_{t_{i}}^{t_{j}}u\cdot dt=\Delta m_{ex,i,j}$$

where $\Delta m_{ex,i,j}$ is a vector that describes the change in the amount of material between time-points $t_{i}$ and $t_{j}$. Hence, the traditional flux analysis method can be transformed by using time-integrated fluxes. For convenience, in the following, the integrated feeding rate between time-points $t_{i}$ and $t_{j}$ will be represented by $F_{i,j}=\int_{t_{i}}^{t_{j}}u\cdot dt$, which is typically also directly measured with balances.

### 2.1. Metabolic Time Integrated Flux Analysis 

MFA allows the calculation of the intracellular fluxes, provided that certain conditions are fulfilled [17]. Using the integrated form, the intracellular material balance can be reformulated to calculate the material transformed by the intracellular pathways, yielding.

(6) $$\begin{array}{cc} S\cdot \Delta m_{in,i,j}+S_{q}\cdot \Delta m_{ex,i,j}=0 & \forall \;i=1..n-1,\;j=i+1..n \end{array}$$

where $\Delta m_{in,i,j}$ is a vector of the transformed material of the intracellular compounds and n is the number of timepoints at which samples were taken. The similarity of Equation (6) to the classical MFA formulation is eminent and thus, we name this approach Metabolic time integrated Flux Analysis (MtiFA).

The change in each of the extracellular compounds can be calculated between $\prod_{k=1}^{n-1}\left(n-k\right)$ combinations of time points. Linear programming is used for solving the system of equations. One advantage is that the error between the measured and estimated extracellular compound can be weighted in case the network exhibits redundancies and is overdetermined. Moreover, the inter-dependences for the extracellular compounds between time-points $t_{i}$ and $t_{j}$ can be accounted for with.

(7) $$\underset{c_{ex}\cdot V,\int_{t_{0}}^{t}u\cdot dt}{\min}\left\{\underset{t}{\sum }{\left\|\frac{c_{ex,mes}\cdot V_{mes}-c_{ex}\cdot V}{w_{t}}\right\|}_{1}+\underset{t}{\sum }{\left\|\frac{F_{mes}-F}{v_{t}}\right\|}_{1}\right\}$$

where $w_{t}$ and $v_{t}$ are the vectors of the weighting values that correspond to the extracellular compounds/cumulative feedings (the index designating that a different value can be used for each measured time-point and concentration/cumulated feed) and ‖·‖ is the absolute error one-norm. The minimization is subject to the intracellular material balance, Equation (6), which must hold for all possible combinations of time-points (note that the combinations are not independent). In addition, constraints accounting for the reversibility of certain fluxes can be implemented and/or bound to the concentrations (e.g., concentrations must be greater or equal to zero) can be specified. Writing the problem in the linear programming form yields:

(8) $$\underset{\epsilon_{c},\;\epsilon_{u}\;}{\min}\left\{\underset{t}{\sum }\frac{\epsilon_{c,t}}{w_{t}}+\underset{t}{\sum }\frac{\epsilon_{u,t}}{v_{t}}\right\}\;s.t.:\;c_{ex,mes}\left(t\right)\cdot V_{mes}\left(t\right)-c_{ex}\left(t\right)\cdot V\left(t\right)\leq \epsilon_{c,t}\;c_{ex}\left(t\right)\cdot V\left(t\right)-c_{ex,mes}\left(t\right)\cdot V_{mes}\left(t\right)\leq \epsilon_{c,t}\;F_{mes}-F\leq \epsilon_{u,t}\;F-F_{mes}\leq \epsilon_{u,t}\;S\cdot \Delta m_{in,i,j}+S_{q}\cdot \left(c_{ex}\left(t_{j}\right)\cdot V\left(t_{j}\right)-c_{ex}\left(t_{i}\right)\cdot V\left(t_{i}\right)-F_{i,j}\right)=0\;\int_{t_{i}}^{t_{j}}v_{irreversible}\cdot X\cdot V\cdot dt\geq 0\;\int_{t_{i}}^{t_{j}}q_{irreversible}\cdot X\cdot V\cdot dt\geq 0$$

The linear programming formulation of the MtiFA was implemented in Matlab 2016a and solved using the Matlab function linprog with default settings (code available as Supplementary Material S1). This method requires the measurement of (exometabolomic) concentrations, as well as measurements of the volume and amount of feeding (typically obtained using balances) in the case of fed-batch processes. All of these measurements are also required for classical MFA. The Matlab implementation of the MtiFA method can be found in the Supplementary Material, S1.

### 2.2. Time-Integrated Flux Balance/Variability Analysis

Tantamount to FBA/FVA, the sum of changes in material from one time-point to the next can be maximized/minimized. In addition to Equation (7), the minimization of the error between the measured and estimated concentrations and feedings, i.e.,

(9) $$\underset{c_{ex}\left(t\right)\cdot V\left(t\right)}{\max or\min}\left\{\overset{n-1}{\underset{i=1}{\sum }}\frac{c_{ex}\left(t_{i+1}\right)\cdot V\left(t_{i+1}\right)-c_{ex}\left(t_{i}\right)\cdot V\left(t_{i}\right)}{d_{i}}\right\}$$

where $d_{i}$ is a weighting factor that can be used to make certain changes more pronounced than others and/or can also be used to balance the maximization/minimization against the minimization of the error. If the solution space of the time integrated Flux Variability Analysis (tiFVA) is to be constrained, e.g., by the optimum obtained from time integrated Flux Balance Analysis (tiFBA), then additional constraints that account for this can be added to the set of constraints (code available as Supplementary Material S2).

### 2.3. Sparse Time Integrated Flux Balance/Variability Analysis

A Sparse FBA has been implemented in the COBRA toolbox [18]. Similarly, to this method, a Sparse tiFBA method can be formulated by linking the unknown changes in mass to integer variables using the big M method:

(10) $$\Delta m_{in,i,j}\geq \varepsilon \cdot Y-M\cdot Z\;\begin{array}{cc} \Delta m_{in,i,j}\leq M\cdot Y-\varepsilon \cdot Z & \forall \;i=1..n-1,\;j=i+1..n \end{array}$$

where M is a large positive value (chosen to be slightly greater than the upper time integrated flux bounds) and ε is a small positive value (set to 10^−3^). Accounting for the fact that the changes can either be positive (entry in Y equals one) or negative (entry in Z equals one) yields:

(11) Y+Z≤1

where Y and Z are vectors of integer variables. In order to promote sparse solutions, the integer variables are added to the objective function in the following way:

(12) $$\underset{Y,Z}{\min}\left\{\sum \gamma \cdot Y+\sum \gamma \cdot Z\right\}$$

where γ is a vector of the weighting factors that balance the sparseness of the solution against the fit (Equation (7)) and maximization/minimization of particular changes (Equation (9)). The original Linear Programming Problem is such transformed into a Mixed Integer Linear Programming Problem. In order to steer the solver towards desired solutions and to reduce the computation times [19], upper ($ub_{I}$) and lower bounds ($lb_{I}$) for the number of integer variables are introduced:

(13) $$\sum Y+\sum Z\leq ub_{I}$$

(14) $$\sum Y+\sum Z\geq lb_{I}$$

The linear programming formulation of the tiFBA was implemented in Matlab 2016a and solved using the Matlab function intlinprog with default settings, except for Integer Tolerance which was set to 10^−6^ and the Constraint Tolerance which was set to 10^−8^. The Matlab implementation of the tiFBA, tiFVA and the corresponding sparse methods can be found in the Supplementary Material, S2.

### 2.4. Simulation Case I

**Figure 1:**
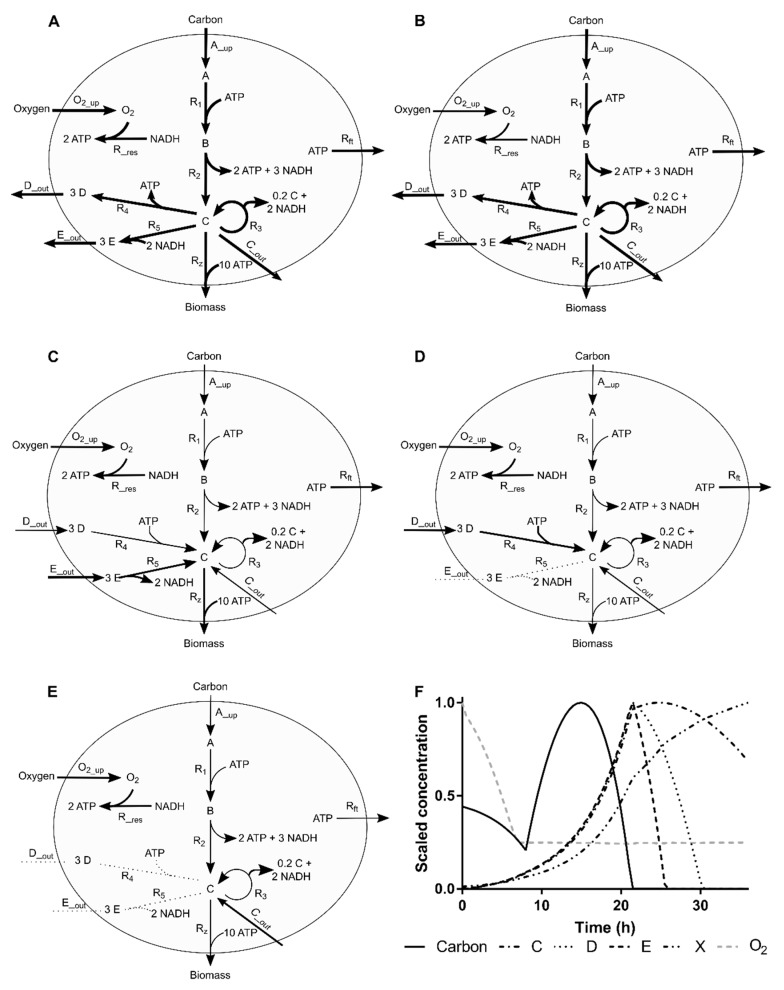
Simulation case network and the evolution of the flux distribution along the simulated process. (**A**–**E**) Arrows width and direction denote reaction flux (wide, normal, thin and doted lines denote large, normal, residual and no flux, respectively). (**F**) Dynamics of scaled extracellular metabolite concentrations along time with different phases highlighted (**A**–**E**).

A simple simulation case was used to evaluate the capabilities of the MtiFA methodology (code available as Supplementary Material S3). The case was adapted from Edward et al. [20]. The network scheme and the evolvement of the flux distributions during the simulated process are presented in Figure 1. Details of the model and simulation can be found in the Appendix A, the Matlab implementation can be found in the Supplementary Material, S3. It can be seen that the simulation case is topologically simple but the time evolution of the flux distribution makes the estimation of the fluxes from measured concentration data challenging. 

#### 2.4.1. Process Operation

The process can be divided into five different regimes. The starting phase is characterized by non-limiting conditions, where both fluxes and metabolite concentrations present high values (Figure 1A). The oxygen concentration is the first metabolite to limit the respective transport flux, thus, lowered flux and concentration were observed in the second phase (Figure 1B), which then impacts the rest of the system (Figure 1C). In this phase, cells start to use E as a complementary carbon source to produce the biosynthetic precursor C (which does not require oxygen). When E is depleted, cells start to import D to circumvent the lack of E and oxygen (Figure 1D). The last phase is characterized by basal fluxes and the import of C to be used as a carbon source (Figure 1E). These variations in fluxes are accompanied by changes in the respective extracellular metabolite concentrations (Figure 1F). The shown extracellular concentrations profiles are representative of many bioprocesses and the changes in the slope make the estimation of the fluxes from measured data challenging. 

#### 2.4.2. Simulated Sampling Strategies and Noise Levels

**Figure 2:**
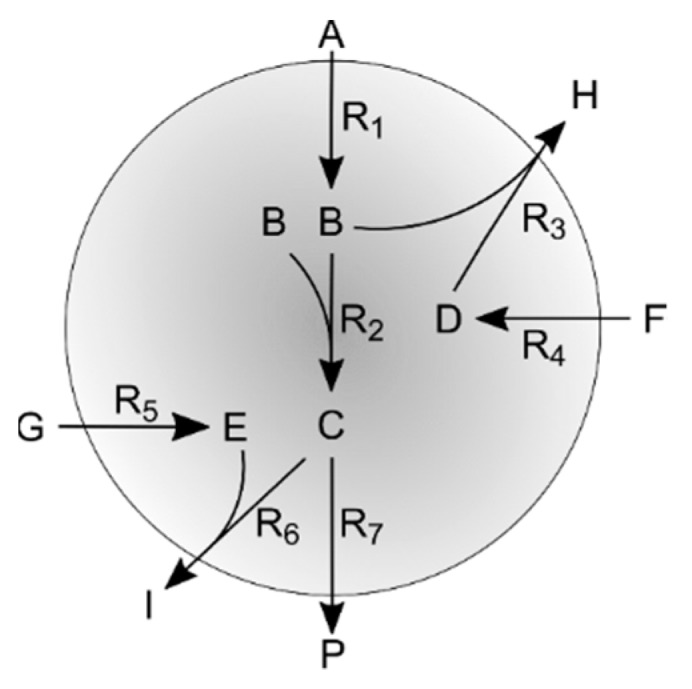
Representation of the second simulation case network.

Two scenarios were investigated, where (1) sufficient concentrations are measured to exactly determine all fluxes with MFA, referred to as the determined case. Namely, the concentrations of A, C, D, E and O2 were considered as measured, allowing the estimation of fluxes A_up, C_out, D_out, E_out and O2_up, respectively, in Figure 1A, and (2) one additional concentration is assumed to be measured (namely production of biomass, Rz in Figure 1A), making the system of equations overdetermined, in the sense of the definition provided by Klamt and co-authors [17], i.e., determined and redundant.

Different sampling strategies and noise levels were simulated, allowing the evaluation of the performance of the MtiFA method under different conditions.

Three sampling strategies were considered: (1) every 5 h, (2) hourly, and (3) normal sampling with four samples being taken per working day, approximately every two hours (i.e., without samples during night). 

Five different strategies for noise level generation were considered. These include 5%, 10% and 15% of average error when compared with the true (simulated) value; a rational error level of 5% for all metabolites concentrations except for biomass and volume measurements where 1% and 2%, respectively, were assumed as more realistic deviations; and lastly, an approach where a metabolite at a time was considered as an outlier. In this case, the rational/normal errors were used with only one difference: a three times greater standard deviation for the outlier metabolite was applied. Such errors were sampled from a normal (with average 0 and standard deviation 1, unless stated otherwise) and beta (with alfa and beta parameters assuming values of 2 and 5; the values were centered after sampling) probability distributions. All combinations of the previous sampling and noise patterns were tested 100 (*k*) times. The average prediction errors (MAE) were calculated between simulated values before adding noise and the concentrations using the MtiFA algorithm.

(15) $$MAE=\frac{\sum_{j=1}^{k=100}\sum_{i=1}^{n}\left|c_{ex,mes}\left(t_{i},\;r_{j}\right)\cdot V_{mes}\left(t_{i},\;r_{j}\right)-c_{ex}\left(t_{i},\;r_{j}\right)\cdot V\left(t_{i},\;r_{j}\right)\right|}{k\cdot n}$$

### 2.5. Simulation Case II

To further assess the impact of network redundancy and constrains defined by irreversibility and weighting factors, a second simple theoretical case study was used. Its network is shown in Figure 2 the model is described in detail in the Appendix B (code available as Supplementary Material S4).

#### 2.5.1. Simulated Sampling Strategies and Noise Levels

The same sampling strategies and noise levels were used as in simulation case I.

#### 2.5.2. Test Cases (Measured and Estimated Fluxes; Irreversible Reactions)

The MtiFA determined case considered reactions 1, 4 and 5 as measured, while for the overdetermined case flux, data for reactions 3, 6 and 7 were added. To test the influence of constrains imposed by irreversible reactions, either no constrains were added or reactions 1–4 were considered to be irreversible.

### 2.6. Experimental Case

An experimental case was used to showcase the capabilities of the sparse tiFBA, i.e., an analysis of Human embryonic kidney 293 (HEK) cultivations. An Ambr 250 was used to perform 24 cultivations in parallel. The cultivations were run in fed-batch mode, and glucose feeding started on day 3 or 4. The same process protocol was used for all runs. One sample was taken each day per cultivation, 12 samples in total per cultivation. The concentrations of Glc, Gln, Amm, Lac, Glu and Pyr were determined using Roche Cedex bio HT, the concentrations of His, Asn, Ser, Arg, Gly, Asp, Thr, Ala, Pro, Orn, Cys, Lys, Tyr, Met, Val, Ile, Leu, Phe, Trp and Urea using a UPLC method with UV derivation and viable cell density (VCD) was measured using Vicell counter for biomass. The experimental approach and data are described in more detail in [21].

In this study, the batch phase was analyzed for 10 cultivation runs in which five different media where assessed, namely media 1 during run 1 to 3 (triplicates); media 2 during runs 4 and 5 (replicates); media 3 during run 6; media 4 during runs 7 and 8 (replicates) and media 5 during runs 9 and 10 (replicates). It needs to be noted that for some of the samples, measurements of some concentrations are not available, i.e., missing at random. The batch phase, days 1 to 3, was selected as (1) it can be assumed that during exponential growth, the cells are in a quasi-steady state, and (2) the metabolism is expected not to change significantly, in particular, the flux direction, as with the proposed tiFBA method, only one flux direction at a time can be considered due to Equation (11). 

A relatively small metabolic network was adopted, which was described previously by Abbate et al. [21]. The network comprises 79 metabolites and 82 reactions. It was assumed that concentrations of Glc, Gln, Lac, Amm, Glu, Pyr, His, Asn, Ser, Arg, Gly, Asp, Thr, Ala, Pro, Orn, Cys, Lys, Tyr, Met, Val, Ile, Leu, Phe, Trp and Urea are measured. The number of measured concentrations is not sufficient to determine all the fluxes in the network uniquely, i.e., the system of equations is underdetermined. The sparse tiFBA approach was applied to determine the time integrated fluxes, assuming that the cells’ objective is to grow as fast as possible. The measured biomass concentration was only used to assess the viability of the predictions.

The fit of the estimated and measured concentrations is assessed using a Weighted Root Mean Square Error (WRMSE):

(16) $$WRMSE=\sqrt{\frac{\sum_{i=1}^{n}{\left(c_{ex,mes}\left(t_{i}\right)-c_{ex}\left(t_{i}\right)\right)}^{2}}{n\cdot \sigma^{2}}}$$

where $\sigma^{2}$ is the variance, which was computed for the experimental concentration values of all the analyzed cultivations.

## 3. Results

### 3.1. Simulation Case I

#### 3.1.1. Quantitative Performance Assessment

**Figure 3:**
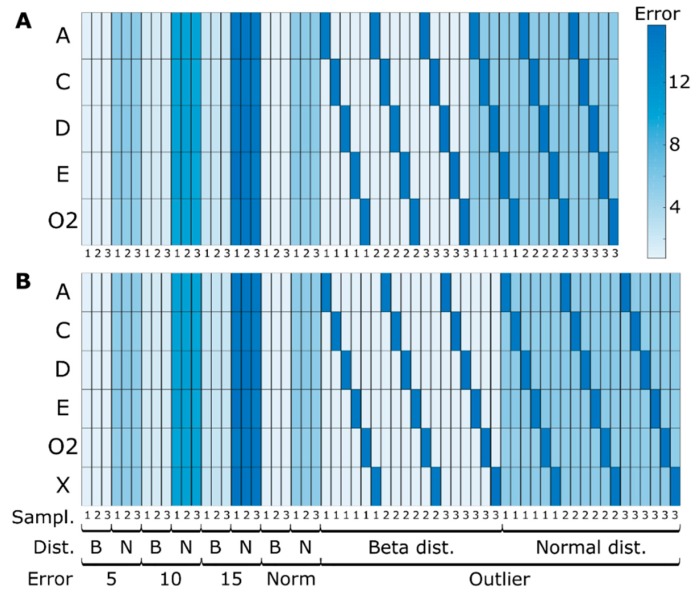
Extracellular metabolites concentration errors when comparing simulated concentrations before adding noise and predicted concentrations with the three different sampling strategies (every 5 h, hourly, typical sampling), different noise levels (5%, 10%, 15% and typical measurement errors—Norm), noise distributions (beta—B and normal—N) for simulation case I using the MtiFA algorithm for (**A**) determined and (**B**) overdetermined case. Each entry represents the average MAE of 100 simulations.

The average prediction errors (MAE) are shown in Figure 3 for the determined and overdetermined cases (panels A and B, respectively). In the determined case, the number of measured concentrations is equal to the number of measured reactions required to estimate the material that is transformed by the intracellular reactions, whereas in the overdetermined case, the number of concentrations is greater than the number of measured reactions.

In all the determined cases, the difference between the target and estimated concentration values are in the range of 10^−15^, which was to be expected as the system is exactly determined. The prediction errors do not vary with changes in the sampling strategy. Even for low sampling frequencies, such as those typically encountered in industrial settings, the MtiFA method is successful in estimating the changes in the concentrations. Classical MFA is sensitive to changes in the sampling strategy, as is well known [12] and as also demonstrated below in Section 3.1.3. Thus, in principle, with the MtiFA method, one can understand pathway usage independent of the sampling strategy, whereas with MFA, the analysis might be corrupted by artifacts introduced during rate estimation. However, MtiFA looks at “total” pathway usage for a specified time interval (the fluxes can vary during this interval), whereas MFA provides information about the conversion velocity for a given moment (or an interval in case it can be considered that the fluxes do not change during the interval). Hence, although MtiFA is not sensitive to the sampling frequency, it does not provide the same information as MFA in terms of time resolution. Outliers also do not seem to significantly impact the estimation performances, as only the metabolites for which outliers were simulated show high prediction errors, but this likely is a network/simulation case-specific observation. The similarity between panels A and B denotes a high agreement between the determined and overdetermined cases, which was per se not expected. The redundancy provided in the overdetermined case seemingly cannot be exploited to improve the prediction accuracy. The consistency of the estimated integrated flux values was checked using the redundancy matrix:

(17) $$0=S_{q}-S\cdot S^{\#}\cdot S_{q}\Delta \cdot m_{ex,i,j}=R\Delta \cdot m_{ex,i,j}$$

where # indicates the pseudoinverse and it was found that the solution is consistent within computer precision (10^−14^). This indicates that the degree of redundancy is not sufficient to significantly improve the predictions. However, it should also be clear that the MtiFA seeks to minimize the absolute error (between measured and estimated masses) and that it will counterbalance the error of the redundant masses, e.g., $\Delta m_{1}=2\cdot \Delta m_{2}$ and when the magnitude of the error in the measured $\Delta m_{mes,2}$ is much lower than that of measured $\Delta m_{mes,1}$, then the algorithm will use $\Delta m_{2}$ to fit $\Delta m_{1}$. In order to further study this tradeoff, simulation case II is introduced.

#### 3.1.2. Qualitative Performance Assessment

**Figure 4:**
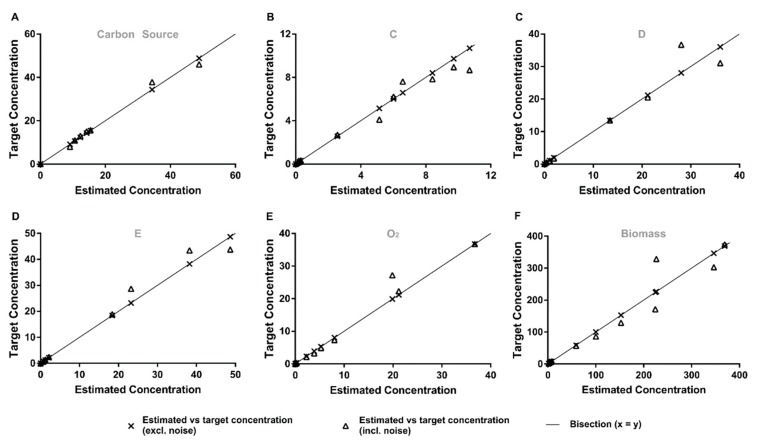
Representative comparison of predicted and target metabolite concentrations for the case where normal distribution, realistic sampling strategy and 15% of added error was used. The plots depict predicted vs. target concentrations of (**A**–**F**) carbon sources C, D, E, O2 and biomass, respectively. Crosses: predicted vs. target concentrations after adding noise; Triangles: predicted vs. target concentrations before adding noise; Line: bisection.

A representative example of the most challenging case, a determined case with few samples and 15% of error in all the variables (A–F), is shown in detail in Figure 4. The proposed method accurately estimates the measured values for the six tested metabolites, although this means that it also fits/includes the measurement noise. It becomes apparent that estimation errors from the concentration of carbon source noise measurements do not propagate to the remaining estimated concentrations. While with the proposed approach, there is no amplification of noise for the estimation of the time integrated fluxes, even with few samples and high measurement noise conditions, it, in some sense, almost “over-fits”. In the future, one could fit models that are linear in the parameters to the mass measurements, such as potentially reducing the over-fit, although also “biasing” the approximation through the introduced model.

#### 3.1.3. Comparison with MFA and Derivative Approach for Flux Estimation

The approach described in Bayer et al. was followed for estimation of the specific fluxes from measured concentration data [22], i.e., (1) The concentration data of the different cases analyzed above were fitted with piecewise polynomial smoothing splines and (2) The fluxes were computed by taking the derivative of the spline with respect to time and dividing by the spline fitted biomass. 

**Figure 5:**
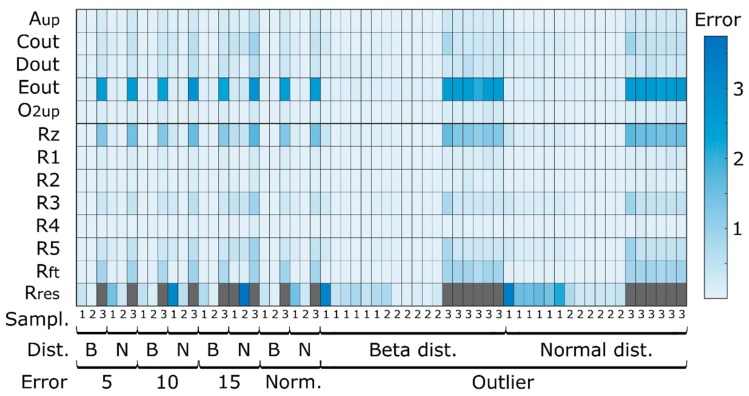
Mean average error between “true” and estimated fluxes for the three different sampling strategies (every 5 h, hourly, typical sampling), different noise levels (5%, 10%, 15% and typical measurement errors—Norm), noise distributions (beta—B and normal—N). To facilitate visualization, outlier values are shown in gray and correspond to values in the range of 15 of MAE. A_up_ to O_2up_ and Rz to Rres represent fluxes estimated based on concentration values and computed by classical MFA, respectively.

Assuming the determined case, the estimated “experimental” fluxes were then used to compute the intracellular fluxes with classical MFA. The MAEs were computed for the difference between the “true” (i.e., the simulated values) and estimated fluxes and are shown in Figure 5. It can be seen that the error values increase significantly for greater levels of noise and a lower number of samples, i.e., noise amplification, as was expected. Outliers also seem to impact on the overall MFA performance, which becomes particularly visible for the typical sampling strategy (Figure 5). Thus, while in MFA, fluxes are assessed, and time integrated fluxes in MtiFA, MtiFA allows an understanding of pathway usage between two timepoints without being impacted by the sample number, measurement noise or outliers in the same way as when one would seek to use MFA (see, e.g., Figure 3). This means that one can also potentially understand overall pathway usage in typical industrial scenarios. Moreover, the MtiFA does not require fitting the experimental concentration data with any approach, which is potentially tedious error-prone work, thus significantly reducing the user effort. However, while the MtiFA can provide insight into the relative use of the pathways and the absolute amount of material transformed by the pathways for a certain time span, it does not provide the rate at which the material is transformed for every time instance (although it might be disputed if the inferred rates, in particular, for few number of samples, represent the true rates). 

### 3.2. Simulation Case II

#### 3.2.1. Effect of Redundancy

**Figure 6:**
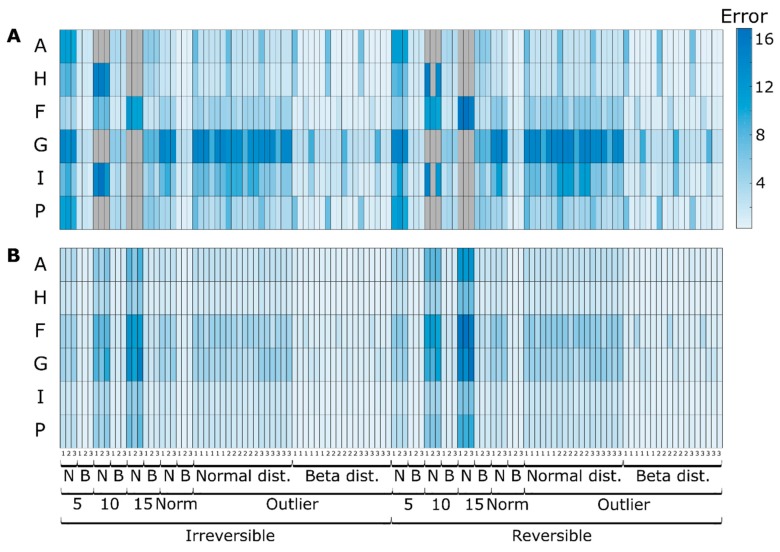
Extracellular metabolites concentration errors when comparing simulated concentrations before adding noise and predicted concentrations for simulation case II with the three different sampling strategies (every 5 h, hourly, typical sampling), different noise levels (5%, 10%, 15% and typical measurement errors—Norm), noise distributions (beta—B and normal—N) for (**A**) determined and (**B**) overdetermined case. To facilitate visualization, outlier values are shown in gray and correspond to values in the range of 40 of MAE. Each entry represents the average MAE of 100 simulations.

Analyzing the determined case results (Figure 6A), two results are noteworthy. Firstly, the metabolite concentration associated with reactions with known dynamics was estimated accurately compared with the originally simulated data. It should be noted that this case is less dynamic than the previous one, with all external metabolites converging to one value (except the fed one). Because of this, the calculated error takes extreme values for low concentration metabolites (e.g., *G*). The second noteworthy result is that depending on the network topology and metabolite concentration, there might be, even if limited in extent, an error propagation effect. This is noticeable on the outlier section between [A, H and P] and [G and I]. Hence, whether error is propagated seems to depend on the properties of the network, as already hypothesized above.

When comparing the determined and overdetermined case results (Figure 6B), we can see the expected increase in accuracy on the dynamics of *I* and remaining values for the bottom part of the network. More noteworthy is the improvement on the evolution of metabolites *P* and *H*. In this case, the estimated results were closer to the true simulated values (before adding noise), showing that the developed methodology can efficiently exploit redundancy to counterbalance the measurement error. 

#### 3.2.2. Effect of Irreversibility Constrains

When more information is provided to the previous example in the form of reaction irreversibility (considering reactions 1–4 irreversible), the estimation results are further improved, see Figure 6. Namely, estimated concentration *A* is closer to the real values, further reducing the impact of the experimental error. More interestingly, this information is propagated on the network to also improve most of the remaining cases to a different degree, depending on the metabolite initial accuracy and the network topology (mainly visible for *F* and *G* cases). Overall, when comparing all the different noise levels, distributions and sampling strategies, more than 85% of the cases showed more accurate results when irreversibility constraints were applied. With classical MFA, irreversibility is per se not considered, but irreversibility constraints can relatively easy be integrated [5].

### 3.3. Experimental Case

**Figure 7:**
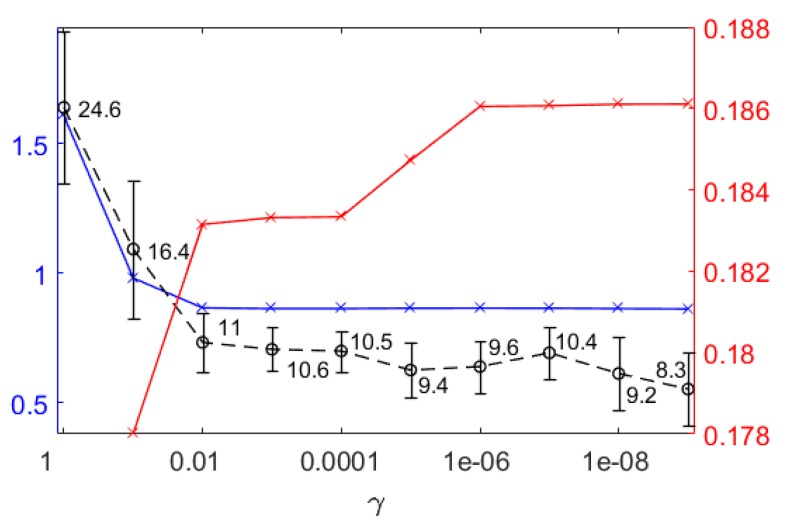
For different values of the γ average, time integrated biomass growth (blue line with x-es), the average number of reactions is set to zero (black dashed line with circles and standard deviations) and cumulated sum of absolute difference between overall estimation errors (red line with x-es) are shown. The cumulated sum of the absolute differences combining all experiments was calculated from the difference between the overall estimation error of all the concentrations of the experiments obtained for two neighboring γ values.

The sparse tiFBA approach allows the definition of the parameter values of the objective function that balance the fit of the experimental values against the number of reactions that are set to zero (γ) as well as the reaction(s) that should maximize $d_{i}$. The parameters for weighting the residuals, $w_{t}$, were set to the value of the standard deviation of the respective concentration, calculated for the measured values of all the runs. Expecting errors in the order of magnitude of 10^−6^, the value for all $d_{i}$ that maximize time integrated biomass growth was heuristically chosen to be 10^−33^, such that growth is a major driving force. γ is an important design parameter balancing the trade-off between the fit and the number of reactions and it needs to be carefully selected. Different values of γ were tested (Figure 7). Two significant increases can be seen, one between  γ values 0.1 and 0.01 and another between 10^−3^ to 10^−6^, otherwise, the values remain fairly constant. The average number of reactions set to zero drops significantly from 24.6 to 11 for γ values of 1 to 0.01, then decreases slightly to 9.4 at γ= 10^−5^ and after a short minor increase to 10.4 at γ=10^−7^, decreases to 8.3 at 10^−9^. For γ values greater than 10^−6^, the fitting of the experimental values is influenced by the number of reactions that are set to zero, which makes the system of equations overdetermined, regardless of the number of missing values. The average integrated biomass growth (blue line, Figure 7) shows a step decrease between 1 and 0.01 and is otherwise constant and not influenced by the number of reactions that are set to zero or the fit of the estimates to the measured values. In order not to influence the predicted biomass growth by setting reactions to zero while still aiming to exploit the surfacing redundancy, a γ value of 0.01 was determined to be ideal and is used in the following steps.

#### 3.3.1. Quantitative Performance Assessment

**Figure 8:**
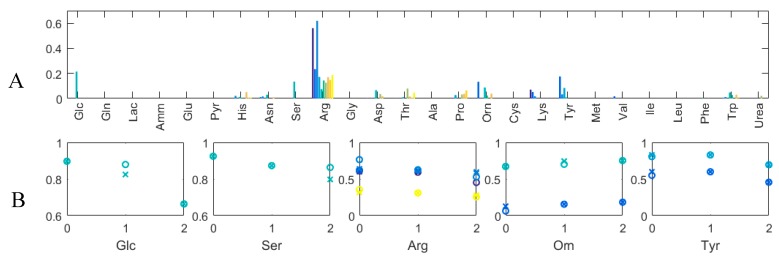
(**A**) Weighted Root Mean Squared Errors (WRMSE) by extracellular compounds for γ= 0.01; (**B**) Normalized concentrations (*y*-axis) over time (days) for those extracellular compounds and Ambr runs with greatest WRMSE. Circles: Measured value; x-es: Estimated Values. Different Ambr runs are shown in different colors and correspond to colors in A.

The WRMSEs by the compounds obtained for γ= 0.01 are shown in Figure 8A. The errors are very low, which was expected, as the system of equations is per se underdetermined and predominantly, the reactions that do not impact the fit of the estimated and measured concentrations are set to zero. A sole error bar can be seen for each Glc and Ser, which are analyzed in more detail in what follows. WRMSE values of the same order of magnitude but for more than three runs can be seen for Arg, Orn and Tyr. The corresponding measured and estimated concentrations over time for the runs with the greatest errors are shown in Figure 8B. Apart from Ser, the other errors seem to stem from rather minor differences between the measured and estimated values. For Ser, a significant difference between the measured and estimated values for day 2 can be seen, the measured Ser value being greater than any of the other measured values. This seems to indicate that this value is an outlier, which is also suggested by the otherwise consistent overall estimation performance of the proposed method. In the case of Arg, the estimated uptake seems systematically lower than the “true” uptake, as the measured concentration values always indicate a greater uptake. Arginine is only required for growth and is available from the medium. The most probable reason for the systematic errors is that the quantity required for biomass is smaller than the one required to explain the data.

**Figure 9:**
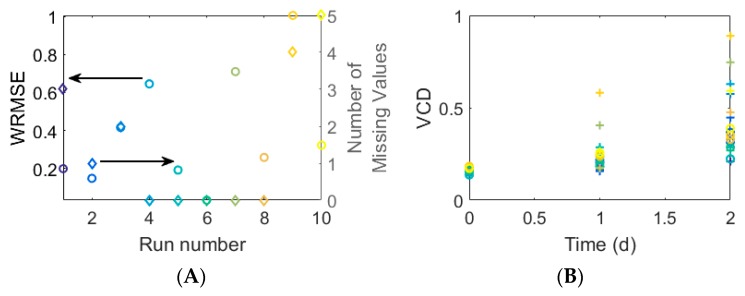
(**A**) The circles which correspond to the left axis show Weighted Root Mean Squared Errors (WRMSE) of predicted biomass concentrations by different Ambr runs. The diamond shapes show the number of missing values per Ambr run. (**B**) Measured and predicted biomass concentrations (circles and x-es, respectively) over time. Different Ambr runs are shown in different colors and correspond to colors in A as well as to those in Figure 8.

The error between the predicted and measured biomass concentrations is shown in Figure 9A along with the number of missing values per Ambr run. For most runs, only one measurement is missing per concentration, but in the case of run 9 and 10, all the measurements of Orn are missing. It seems that the prediction accuracy is not being systematically influenced by missing values (Figure 9A). Overall the biomass prediction performance is not great, which is likely a result of the assumed biomass composition. This lack in performance can also be confirmed when looking at the biomass concentration estimates over time (Figure 9B), where the values predominantly increase between day 1 and 2 and largely do not correspond well with the measured values. One could change the bounds on the biomass growth reaction, introduce an additional constraint to make the growth values between day 1 and 0 as well as 2 and one more similar or change the values of $d_{i}$ to get a closer fit for day 1, but this will not change the overall predicted biomass concentration. 

#### 3.3.2. Qualitative Performance Assessment

**Figure 10:**
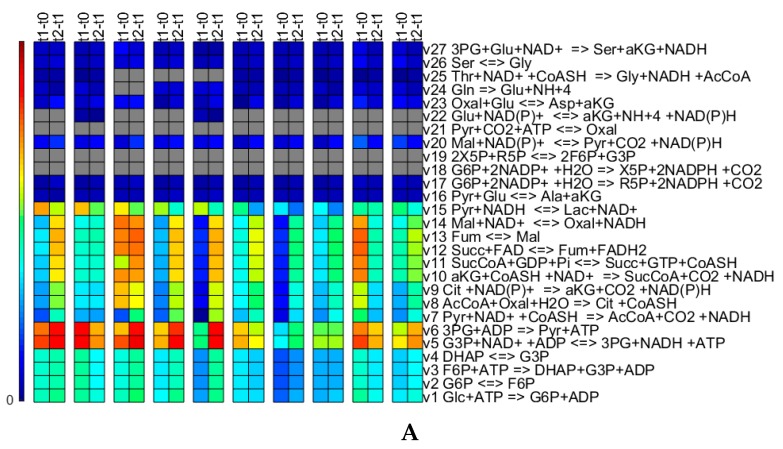

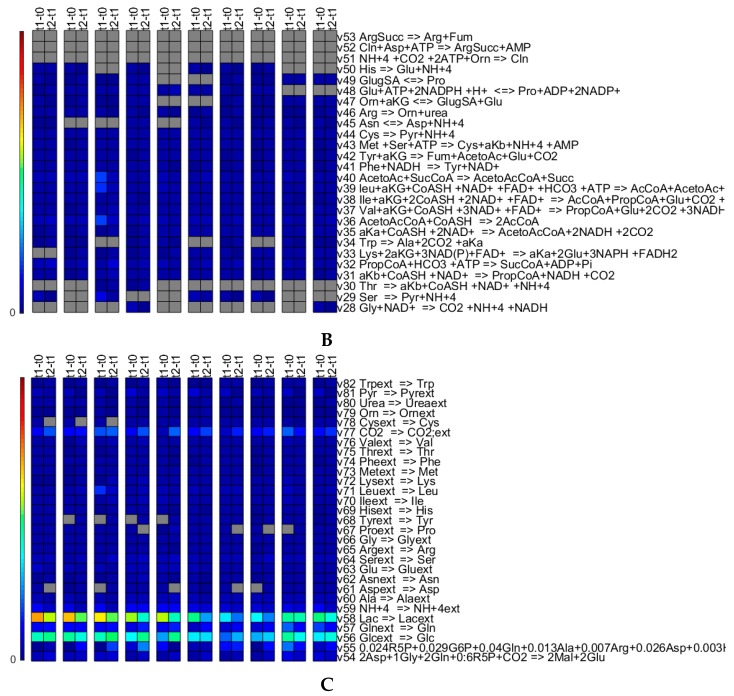
Time integrated fluxes normalized by the biomass concentration values of the respective upper time point for all fluxes of the metabolic network for runs 1 to 10 from the left. The values shown in grey are exactly zero. The colors correspond to the amount of material that is transformed by the reaction. Panel (**A**) shows Glycolysis, TCA cycle, Lactic Acid production, Pentose Phosphate pathways and some part of the amino acid biosynthesis and conversion; (**B**) shows predominantly amino acid biosynthesis and conversion; and (**C**) shows predominantly exchange reactions.

Figure 10 shows biomass concentration normalized transformed masses for all the reactions of the metabolic network obtained for γ= 0.01. Overall, the reactions that are set to zero seem consistent across the runs, and where the reactions are non-zero, the flux values are typically fairly low. The reactions which are set to zero also seem to make sense from a biological point of view. Arg is available from the medium and therefore, does not need to be synthesized (reactions 51 to 53). The switch-off of the pentose phosphate pathway reactions, 18 and 19, can be explained as there is no need for these two reactions to ensure growth (in the case of this network) indeed, only R5P is required for biomass growth and is produced using reaction 17. Reaction 21 is an anaplerotic reaction important for gluconeogenesis, which is normally not active in normal growth conditions on glucose, as also observed here. Reaction 22 is only zero for some cases. This reaction links the TCA cycle with the nitrogen assimilation, although nitrogen (ammonia) can only be produced. Therefore, if in the experimental data there is no need to explain an ammonia consumption, this reaction will be set to zero. It is difficult to explain why reactions 28, 29 and 30 are set to zero, as these are simplified reactions that represent multiple “real” reactions involved in amino acid metabolism. Simulations with a γ value of 10-6 change the pattern slightly in that e.g., the reactions of the pentose phosphate pathways (reactions 19 and 20) are turned on for some runs. This means that depending on the objective of the study (and results), the γ value (and potentially, the other optimization parameters $d_{i}$ and the residual scaling parameters $w_{t}$) might have to be identified independently for every run investigating in detail the fluxes through the network or that the reactions which are set to zero should be enforced to be consistent for all runs in case the focus is on finding an overall network that represents all runs. The latter could be ensured by including all runs in the optimization. 

Comparing the triplicate runs 1 to 3, it strikes that run 3 shows significantly increased flux values for reactions 8 to 13 though slightly lower values for reaction 14. The third run exhibits a significantly lower concentrations profile for Asp [23], which might, in combination with the slight increases in the fluxes of reactions 23, 30 and 40, explain the difference. In case of the replicate runs 9 and 10, the trends between the fluxes of t1-t0 and t2-t1 are reversed. Run 9 exhibits profiles of slightly lower concentrations for Asn, Thr, Pro and Lys, although these profiles seem almost parallel to the ones of run 10. The profiles of concentrations His, Thr, Met, Val, Ile, Leu, Phe and Trp diverge and exhibit an increasing difference between run 9 and 10 towards day 2 [23]. However, all of these differences seem to fall within typically encountered biological and analytical variation.

Comparing the five different media on the basis of the flux patterns (runs 1, 2, 3 to 4, 5 to 6 to 7, 8 and 9, 10), major differences can be seen in glucose uptake and lactate secretion (Figure 10C) and accordingly, in glycolysis and the TCA cycle (Figure 10A). The lower activity in the glycolysis and TCA cycle fluxes in case of media 3, run 6, is also translated into lower biomass growth. Medium 5 shows high glycolysis and TCA cycle fluxes, relatively low lactate secretion and the highest growth rate, properties searched for during media development.

## 4. Conclusions

Overall, the analysis of time integrated fluxes, i.e., the analysis of transformed material, provides about the same understanding as more classical flux analysis. However, in contrast to e.g., classical MFA, the MtiFA does not provide the rate at which the material is transformed for every time instance. Instead, MtiFA provides insight into the relative use of the pathways and the absolute amount of material transformed by the pathways for a certain time span. Furthermore, the proposed methodology offers capabilities that make it very attractive for industrial use, namely:

(1) Few samples are required, i.e., the method is well conditioned for the analysis of sparse and noisy data sets, which is the case of most industrial data.

(2) Since the estimation of the specific fluxes from concentration data is avoided, no assumptions regarding their behavior have to be made. This also means that the proposed method can, in principle, be directly adopted to describe the entire experiment.

(3) The workload and workflow of the proposed method are significantly reduced in comparison to the classic flux analysis methods and the chance of introducing a bias through the estimation of the specific fluxes from the measured concentrations is eliminated. 

## Supplementary Materials

Supplementary materials can be found at https://www.mdpi.com/2076-2607/7/12/620/s1.

## Appendix A. Simulation Case I

### Appendix A.1. Network Topology

The metabolic network (Figure 1A) for the simulation case was adapted from [20]. A carbon source is used to produce biomass as main product and three byproducts, C, D and E. Internally, oxygen is used as electron acceptor to convert redox potential into ATP. Two futile reactions (R_ft_ and C_out_) were added to consider the inherent consumption of ATP and export of biosynthetic precursor C to mimic the energy expended in cellular maintenance and basal membrane permeability, respectively. The stoichiometric matrix reads:

(A1) $$S=\left[\begin{array}{ccccccccccccc} 0 & 0 & 0 & 0 & -1 & 0 & 0 & 0 & 1 & 0 & 0 & 0 & 0\\ 0 & 0 & 0 & 0 & 1 & -1 & 0 & 0 & 0 & 0 & 0 & 0 & 0\\ -1 & 0 & 0 & -1 & 0 & 1 & -1 & -1 & 0 & 0 & 0 & 0 & -1\\ 3 & -1 & 0 & 0 & 0 & 0 & 0 & 0 & 0 & 0 & 0 & 0 & 0\\ 0 & 0 & -1 & 0 & 0 & 0 & 0 & 0 & 0 & 0 & 0 & 0 & 0\\ 0 & 0 & 0 & 0 & 0 & 0 & 0 & 0 & 0 & 0 & -1 & 1 & 0\\ 1 & 0 & 0 & 0 & -1 & 2 & 0 & 0 & 0 & -1 & 2 & 0 & -10\\ 0 & 0 & 0 & 0 & 0 & 3 & 10 & -2 & 0 & 0 & -1 & 0 & 0 \end{array}\right]$$

With reactions R4, D_OUT_, E_OUT_, C_OUT_, R1, R2, R3, R5, A_UP_, R_RT_, R_RES_, O_2_UP_ and R_Z_, and metabolites A, B, C, D, E, O_2_, ATP and NADH, respectively.

### Appendix A.2. Simulated Kinetics

The fluxes that cross the cell boundary were approximated by Michaelis-Menten type kinetics (as this kinetics are typically used to describe e.g., growth/substrate uptake in macroscopic material balances), where carbon source and oxygen were assumed as unidirectional, and the remaining target fluxes were calculated by classic MFA. The extracellular balances depended on their specific production and dilution rate.

For the transport fluxes *J_D_OUT_*, *J_E_OUT_*, *J_C_OUT_*, *J_A_UP_* and *J_O2_UP_*, kinetic rate functions were defined

(A2) $$J_{D_{OUT}}=\frac{V_{Max\_D\_OUT}\cdot A_{OUT}}{A_{OUT}+k_{D\_OUT}}-\frac{V_{Max\_D\_IN}\cdot D_{OUT}}{D_{OUT}+k_{D\_IN}}\cdot \frac{0.02}{A_{OUT}}$$

(A3) $$J_{E_{OUT}}=\frac{V_{Max\_E\_OUT}\cdot A_{OUT}}{A_{OUT}+k_{E\_OUT}}-\frac{V_{Max\_E\_IN}\cdot E_{OUT}}{E_{OUT}+k_{E\_IN}}\cdot \frac{0.05}{A_{OUT}}$$

(A4) $$J_{C_{OUT}}=\frac{V_{Max\_C\_OUT}\cdot A_{OUT}}{A_{OUT}+k_{C\_OUT}}-\frac{V_{Max\_C\_IN}\cdot C_{OUT}}{C_{OUT}+k_{C\_IN}}\cdot \frac{0.001}{A_{OUT}}$$

(A5) $$J_{A_{UP}}=\frac{V_{Max\_A}\cdot A_{OUT}}{A_{OUT}+k_{A}}$$

(A6) $$J_{O_{2\_UP}}=\frac{V_{Max\_O2\_UP}\cdot O_{2\_OUT}}{O_{2\_OUT}+k_{O2}}$$

where *A_OUT_*, *C_OUT_*, *D_OUT_*, *E_OUT_*, *O_2_OUT_* represent the metabolites concentration in the broth and *V_Max_r_* and *k_r_* represent the assumed Michaelis-Menten kinetic parameters for each reaction r and direction (in or out for forward or reverse reaction, if applicable).

The remaining fluxes were computed by classic Metabolic Flux Analysis:

(A7) $$J_{IN}=-S_{IN}^{\#}\cdot S_{OUT}\cdot J_{OUT}$$

With *J_IN_* and *J_OUT_* fluxes of internal and transport reactions, respectively.

The extracellular material balances were defined as:

(A8) $$\frac{dD_{OUT}}{dt}=J_{D\_OUT}\cdot x-D\cdot D_{OUT}$$

(A9) $$\frac{dE_{OUT}}{dt}=J_{E\_OUT}\cdot x-D\cdot E_{OUT}$$

(A10) $$\frac{dC_{OUT}}{dt}=J_{C\_OUT}\cdot x-D\cdot C_{OUT}$$

(A11) $$\frac{dO_{2\_OUT}}{dt}=-J_{O2\_OUT}\cdot x-D\cdot O_{2\_OUT}+kla\cdot \left(O_{2\_S}-O_{2\_OUT}\right)$$

(A12) $$\frac{dA_{OUT}}{dt}=-J_{A\_OUT}\cdot x-D\cdot \left(A_{OUT}-A_{Feed}\right)$$

(A13) $$\frac{dx}{dt}=-J_{Z}\cdot x-D\cdot x$$

(A14) $$\frac{dV}{dt}=u_{Feed}$$

With initial volume *V*, feeding rate *u_Feed_* for the carbon source and dilution rate, *D* = *u_Feed_*/*V*.

Simulation was run with conditions described in Table A1 for parameters and initial conditions.

**Table A1:** List of parameters and initial conditions used for the simulation case I.

Variable/Parameter	Value	Comment
V (initial)	1 L	initial conditions
x (initial)	2	
$O_{2\_OUT}$ (initial)	0.004	
$A_{OUT}$ (initial)	15	
$A_{Feed}$	200	feed characterization
$u_{Feed}$	0.05 L/h	After 8 h of fermentation
		PARAMETERS
$O_{2\_S}$	0.005	Controlled at 20%
$V_{Max\_D\_OUT}$	0.05	
$k_{D\_OUT}$	0.1	
$V_{Max\_D\_IN}$	0.04	
$k_{D\_IN}$	0.1	
$V_{Max\_E\_OUT}$	0.07	
$k_{E\_OUT}$	0.1	
$V_{Max\_E\_IN}$	0.06	
$k_{E\_IN}$	0.1	
$V_{Max\_C\_OUT}$	0.01	
$k_{C\_OUT}$	0.1	
$V_{Max\_C\_IN}$	0.04	
$k_{C\_IN}$	0.2	
$V_{Max\_A}$	0.2	
$k_{A}$	0.1	
$V_{Max\_O2\_UP}$	0.01	
$k_{O2}$	0.0028	

## Appendix B. Simulation Case II

### Appendix B. Network Topology and Simulation Kinetics

An initial metabolite (A) is used to produce two intermediates (B and C), Figure 2. These metabolites (B and C) can be diverted to secondary pathways that use other external resources (F and G, respectively) to produce some byproducts (H and I, respectively) each with one intermediate metabolite (D and E, respectively). In addition, there is one final byproduct (P) on the main pathway. Overall there are seven reactions and ten metabolites, four of which are internal metabolites. The stoichiometric matrix for this simulation case reads:

(A15) $$S=\left[\begin{array}{ccccccc} 1 & -2 & -1 & 0 & 0 & 0 & 0\\ 0 & 1 & 0 & 0 & 0 & -1 & -1\\ 0 & 0 & 0 & 0 & 1 & -1 & 0\\ 0 & 0 & -1 & 1 & 0 & 0 & 0 \end{array}\right]$$

With reactions R_1_ to R_7_ and internal metabolites B, C, E and D, respectively.

The kinetics are driven by concentrations of A, F and G, via reactions R_1_, R_4_ and R_5_. For the import fluxes (reactions 1, 4 and 5), kinetic rate functions were defined:

(A16) $$J_{1}=\frac{0.4\cdot A}{A+0.1+5\cdot H^{2}}$$

(A17) $$J_{4}=\frac{0.2\cdot F}{F+4.5}\cdot \frac{A}{A+0.0001}$$

(A18) $$J_{5}=\frac{0.1\cdot G}{G+10.5}\cdot \frac{A}{A+0.0001}$$

where A, F, G and H represent the metabolites concentration in the broth and *V_Max_r_* and *k_r_* Michaelis-Menten kinetic parameters are listed as numerical values in the equation for each reaction. The initial metabolite concentrations were considered to be 50, 7, 6 and 0.1 AU for A, F, G and I. The remaining initial metabolite concentrations were zero.

The remaining fluxes were computed by classic Metabolic Flux Analysis:

(A19) $$J_{IN}=-S_{IN}^{\#}\cdot S_{OUT}\cdot J_{OUT}$$

With *J_IN_* and *J_OUT_* fluxes of internal and transport reactions, respectively. The model was simulated for 50 h.
